# Novel Genomic Regions of *Fusarium* Wilt Resistance in Bottle Gourd [*Lagenaria siceraria* (Mol.) Standl.] Discovered in Genome-Wide Association Study

**DOI:** 10.3389/fpls.2021.650157

**Published:** 2021-05-07

**Authors:** Yanwei Li, Ying Wang, Xinyi Wu, Jian Wang, Xiaohua Wu, Baogen Wang, Zhongfu Lu, Guojing Li

**Affiliations:** Institute of Vegetables, State Key Laboratory for Quality and Safety of Agro-Products, Zhejiang Academy of Agricultural Sciences, Hangzhou, China

**Keywords:** bottle gourd, *Fusarium* wilt, genome-wide association (GWAS), novel genomic regions, qRT-PCR

## Abstract

*Fusarium* wilt (FW) is a typical soil-borne disease that seriously affects the yield and fruit quality of bottle gourd. Thus, to improve resistance to FW in bottle gourd, the genetic mechanism underlying FW resistance needs to be explored. In this study, we performed a genome-wide association study (GWAS) based on 5,330 single-nucleotide polymorphisms (SNPs) and 89 bottle gourd accessions. The GWAS results revealed a total of 10 SNPs (*P* ≤ 0.01, −log_10_*P* ≥ 2.0) significantly associated with FW resistance that were detected in at least two environments (2019DI, 2020DI, and the average across the 2 years); these SNPs were located on chromosomes 1, 2, 3, 4, 8, and 9. Linkage disequilibrium (LD) block structure analysis predicted three potential candidate genes for FW resistance. Genes *HG_GLEAN_10001030* and *HG_GLEAN_10001042* were within the range of the mean LD block of the marker BGReSe_14202; gene *HG_GLEAN_10011803* was 280 kb upstream of the marker BGReSe_00818. Real-time quantitative PCR (qRT-PCR) analysis showed that *HG_GLEAN_10011803* was significantly up-regulated in FW-infected plants of YD-4, Yin-10, and Hanbi; *HG_GLEAN_10001030* and *HG_GLEAN_10001042* were specifically up-regulated in FW-infected plants of YD-4. Therefore, gene *HG_GLEAN_10011803* is likely the major effect candidate gene for resistance against FW in bottle gourd. This work provides scientific evidence for the exploration of candidate gene and development of functional markers in FW-resistant bottle gourd breeding programs.

## Introduction

Bottle gourd [*Lagenaria siceraria* (Mol.) Standl.] (2*n* = 2 × = 22), also known as calabash or long melon, is a member of the *Legendaria* genus, Cucurbitaceous family, and is an annual plant (Whitaker, 1971; Erickson et al., 2005). Its fresh young fruits are eaten as a vegetable in parts of Asia and Africa, while dry old fruits are used for containers, musical instruments, and crafts (Heiser, 1979; Morimoto and Mvere, 2004). Moreover, due to its strong resistance to disease and abiotic stress, bottle gourd is commonly used as grafting rootstock for other crops, such as watermelon and melon (Lee, 1994; Yetisir and Sari, 2003). According to records, bottle gourd has been cultivated in China for more than 7,000 years, covering a cultivation area of more than 2 million mu; this is an important vegetable crop in China, especially in the southern part.

*Fusarium* wilt (FW), which is caused by *Fusarium oxysporum*, is a typical soil-borne disease of economic crops worldwide (Katan, 1994; Wechter et al., 2012; Bodah, 2017). Since this pathogen can survive in the absence of host-infected plants, once the disease occurs in the field, *F. oxysporum* is likely to remain in the soil indefinitely, which seriously affects the yield of crops (Cha et al., 2016; Khan et al., 2017). FW has a broad host on cucurbit crops, including watermelon, melon, cucumber, luffa, and bottle gourd (Bodah, 2017). Usually, the infected plants morphologically show a constriction in the stem xylem, resulting in vascular bundle clogging, plant wilting, or death (Freeman et al., 2002; Singh et al., 2017). FW commonly occurs during the whole growth period of bottle gourd, especially the flowering to fruiting period. Its incidence is approximately 20–40%, and severe cases could cause devastating losses (data from Ningbo Institute of Agriculture), which severely restrict the sustainable development of the bottle gourd industry.

Breeding resistant varieties is one of the most effective and economic methods to control FW disease. At present, a series of commercial varieties that are highly resistant to FW has been grown for production, such as watermelon, melon, and cowpea (Zink and Gubler, 1985; Martyn and Bruton, 1989; Ehlers et al., 2000, 2009). To improve FW resistance, we need to exploit markers tightly linked to FW resistance using quantitative trait loci (QTL) and then generate germplasm by molecular marker assistant selection (MAS; Zhao et al., 2014; Li et al., 2017). To date, QTLs/genes conferring FW resistance have been thoroughly studied in many crops. For example, a dominant gene *I-2* that confers resistance to race 2 of FW was cloned in tomato by map-based cloning (Simons et al., 1998; Catanzariti et al., 2015). Using the same map-based cloning technique, Joobeur et al. (2004) identified two candidate genes of melon FW resistance in the physical range of Fom-2. Several genes associated with cowpea FW resistance were identified using QTL analysis in “California Blackeye 27” (Pottorff et al., 2012, 2013). In cucumber, a major QTL Foc2.1 conferring resistance to FW was detected using recombinant inbred lines (Zhang et al., 2014), and a major QTL, Fo-1.1, associated with FW resistance to race 1 was identified by using selective genotyping in genetic populations derived from related watermelon lines (Lambel et al., 2014). However, research progress on the FW resistance of bottle gourd is relatively limited. Only its specialization of *F. oxysporum* f. sp. *lagenariae* has been reported, whereas the genetic mechanism of FW resistance and related genes/QTLs are unknown in bottle gourd.

To date, a high-density genetic map has been constructed, and a series of ISSR, SSR, and single-nucleotide polymorphism (SNP) markers has been exploited for bottle gourd (Xu et al., 2011, 2014; Bhawna et al., 2014), allowing the establishment of various marker–trait associations, such as association analysis for the free glutamate content of bottle gourd (Wu et al., 2017). Genome-wide association study (GWAS), based on linkage disequilibrium (LD), has also been widely used in the study of plants, and various results have been reported (Joobeur et al., 2004; Wang et al., 2009; Sabbavarapu et al., 2013; Zhang et al., 2014). In bottle gourd molecular breeding, Wu et al. (2017) performed a GWAS for SNPs related to the free glutamate content of the umami factor. With the development of quantitative genetics, many researchers have proposed different analytical models, such as efficient mixed-model association (Kang et al., 2008), compressed mixed linear model (Zhang et al., 2010), restricted two-stage multi-locus GWAS (He et al., 2017), etc. Among them, general linear model (GLM) and mixed linear model (MLM) are still the common GWAS methods in plants (Huang et al., 2010; Li et al., 2013; Fang et al., 2017). In this study, we initially genotyped 89 bottle gourd accessions using 5,330 SNPs and surveyed the disease index (DI) of FW resistance in two consecutive years. We then performed a GWAS to identify significant associated SNPs and potential candidate genes. Finally, three candidate genes associated with FW resistance were verified by quantitative real-time PCR (qRT-PCR). Our study is the first to use GWAS to identify genomic regions and candidate genes associated with FW resistance. The GWAS results can lay a foundation for MAS breeding and the genetic mechanisms of FW resistance in cucurbit crops.

## Materials and Methods

### Plant Materials

Germplasm consisting of 89 bottle gourd accessions was collected, consisting of 87 accessions from wide areas across China, one accession from Europe, and one accession from Mexico (Supplementary Table 1). All accessions (inbred lines) were evaluated for FW resistance in a glasshouse of the Haining Experimental Station (30° N, 120° E) in 2019 and 2020. According to a completely randomized block design, the plants were studied based on two replications in both years.

### Inoculation System of FW Resistance in Bottle Gourd

During 2018–2019, bottle gourd FW fungus was isolated from wilted plants that were collected from severely affected areas such as Shaoxing and Haining (Supplementary Figures 1A,B). According to the conventional tissue separation method, FW strains with obvious antagonistic effects were obtained by using potato dextrose agar (containing 100 mg/ml Kana and Amp) for screening four to five times (Supplementary Figure 1C). Under a microscope with 10 × 40 magnification, small conidia were observed to be ovoid or kidney-shaped, and large conidia were spindle-shaped or sickle-shaped with hooked apex (Supplementary Figure 1D). PCR assays showed that the similarity between the sequence of FW isolates and the 16S rRNA sequence was as high as 99%. After cytological tests and PCR detection, the isolates were identified as *F. oxysporum* f. sp. strains and were stored at 4°C at the Zhejiang Academy of Agricultural Sciences, Hangzhou, China.

Each FW strain was shake-cultured on potato sucrose broth for 3 days in the dark at 28°C at 200 rpm. With the use of a hemacytometer, the conidial suspension was adjusted to a final concentration of 1.0 × 10^6^ conidia/ml with sterile distilled H_2_O. The seeds of each accession were sown in mixed soil (nutritional soil/vermiculite = 3: 1) in plastic pots (6 by 6 by 5 cm) and were grown in a glasshouse set at 24°C, 16-h light/18°C, 8-h darkness, 60% humidity. At the second true leaf of the seedling spreading stage, we used the root dipping method for bottle gourd FW resistance screening and testing. Each accession consisted of 10–12 seedlings, and two duplicates were set per environment.

### Disease Assessment and Statistical Analysis

Leaf damage was used as a main index to evaluate resistant/susceptible phenotypic traits. The standard reported by Gao et al. (2015) and Xu et al. (2016) was further improved and implemented with a few modifications. We classified the phenotypes of plants according to a 0–4 scale as follows: level 0, no disease symptoms, i.e., immune (I); level 1, slight disease symptoms, featured by less than 25% of leaves with disease symptoms, with normal plant growth, i.e., highly resistant (HR); level 2, slight wilt symptoms, featured by 25–50% of leaves with disease symptoms, i.e., resistant (R); level 3, moderate wilt symptoms, featured by 50–90% of leaves with disease symptoms, i.e., susceptible (S); and level 4, completely wilted or dead plants, i.e., highly susceptible (HS; Supplementary Figure 2). After 10–12 replicates per material were evaluated individually, we calculated the mean value to determine the disease severity for each accession. The DI was calculated according to the following equation:

$$DI=\frac{\sum \text{P}i\times \text{n}i}{N\times \text{P}4}\times 100\%,$$

where DI is the disease index, P*i* is the grade of the DI, n*i* is the plant number of the corresponding DI grade, *N* is the total number of plants investigated, and P*4* is the highest DI grade.

According to the DI scores, the FW resistance of each material was determined following Shen and Li (2008) with a few modifications: immune (DI = 0, level 0), highly resistant (0 < DI ≤ 15%, level 1), resistant (15% < DI ≤ 45%, level 2), susceptible (45% < DI ≤ 75%, level 3), and highly susceptible (DI > 75%, level 4).

### SNP Genotyping, LD, and Population Structure

The SNP markers used in this study were generated from RAD sequencing with paired-end sequencing (150 bp) on the Illumina HiSeq platform. We initially found 89 bottle gourd accessions that aligned to the Hangzhou gourd reference genome of ∼330 Mb (Wang et al., 2018)^1^ and then removed those SNPs with a minor allele frequency (MAF) of ≤0.01 and a heterozygous rate ≥25% for data filtering. This left a total of 6,222 high-quality SNPs. Of these SNPs, 85.66% were located on the 11 chromosomes of the bottle gourd, leaving 5,330 high-quality SNPs. These were used for the correlation analysis of traits (Wu et al., 2017). The density of SNP markers was estimated to be one SNP per 59.37 kb for the 11 bottle gourd chromosomes.

The LD parameters (*r*^2^) for estimating the LD distance of the genome between pairwise SNPs (MAF > 0.01) were calculated using PLINK V1.07 (Purcell et al., 2007; Xu et al., 2021, unpublished), and the average LD decay was drawn with R. The population structure was constructed by STRUCTURE 2.3.4 software (Evanno et al., 2005). *K* (number of subgroups) values were set to 2–8, with 10,000 (MOMC, Markov chain Monte Carlo)–100,000 runs (MCMC, Monte Carlo Markov Chain) with four replications. Then, the best value of *K* was determined by *Ln P*(*D*) and *Delta K* according to the principle of maximum likelihood (Evanno et al., 2005). The neighbor-joining tree was constructed using PHYLIP software. The kinship matrix was assessed based on the SNP dataset using TASSEL 5.2.14 to determine the relatedness among individuals (Anderson and Weir, 2007; Zhang et al., 2010). In previous studies, the population was divided into two subgroups depending on the markers used in the tests (Wu et al., 2017).

### Genome-Wide Association Analysis and LD Block Construction

For natural populations, the population structure and relative kinship always lead to high levels of false positives in association maps (Yu et al., 2006). After assessment of the population structure (*Q*), relative kinship (*K*), and principal component analysis (PCA) of 89 accessions, four correlation analysis models including (1) a general linear model GLM (*Q*), GLM (PCA) and (2) a mixed linear model MLM (*Q* + *K*), MLM (PCA + *K*) were used to conduct a genome-wide correlation analysis of FW resistance using TASSEL 5.2.14 (Anderson and Weir, 2007; Zhang et al., 2010). The significance threshold for SNP–trait associations was determined by 1/*n*, where *n* is the number of markers in the association panel (Yang et al., 2014), and *P* ≤ 1/5,330 or −log_10_*P* ≥ 3.7. Considering that population structure and kinship reduced the detection efficiency of SNPs associated with FW resistance, the −log_10_*P* value of significantly associated SNPs identified in this study was low, which has also appeared in previous studies (Atwell et al., 2010; Huang et al., 2010). In order to fully exploit the valuable genetic information in the bottle gourd germplasm population, the significant threshold for SNP–trait associations was set as −log_10_*P* = 2. This threshold has already been applied to other traits in an association analysis (Li et al., 2015; Zhang et al., 2018). The correlation analysis results were plotted using a Manhattan plot and *Q*–*Q* plot based on the “qqman” package in R software.

The genome-wide LD decay rate, defined as the LD block distance where the LD decays to half of its maximum value, was about 400 kb in a natural population of bottle gourd (from Xu et al., 2021, unpublished). We defined the average LD decay distance as the candidate region to select candidate genes associated with large-effect SNPs. The genome of “Hangzhou gourd” was used as a reference sequence (Wang et al., 2018). Based on the genomic annotations of GourdBase,^2^ potential candidate genes for FW resistance were predicted.

### Validation of Candidate Genes

The expression levels of the candidate genes were measured before and after infecting plants with FW by using qRT-PCR. Based on the phenotype data in 2019 and 2020, Hanbi (HR to FW, level 1), Yin-10 (HR to FW, level 1), and YD-4 (HS to FW, level 4) were chosen as extreme materials and were cultivated in the glasshouse. The leaves from healthy plants (CK) and treatment plants were collected 3 days after FW infection and stored in liquid nitrogen. Total RNA was extracted from Hanbi, Yin-10, and YD-4 leaves using an RNA Simple Total RNA kit (Tiangen, China). After the quality and concentration of total RNA were evaluated using 1% agarose gel and an Agilent 2100 Bioanalyzer, complementary DNA (cDNA) was synthesized by using a Script cDNA Kit (Tiangen, China) with a standard protocol. The CDS sequences of genes were obtained from the GourdBase website.^3^ qRT-PCR primers (Supplementary Table 2) were designed using the Integrated DAN Technologies website^4^ and were synthesized by Sangon Biotech (Shanghai) Co., Ltd. The bottle gourd *TuB-α* gene (*BG_GLEAN_10019523*) was used as the internal control gene. qRT-PCR was performed on a Bio-Rad CFX96 Touch q-PCR System (Bio-Rad, CA, United States) with SuperReal PreMix Plus/SYBR Green (Tiangen, China). Each reaction was replicated three times. The relative expression level of candidate genes was evaluated by the 2^–ΔΔ*Ct*^ method (Livak and Schmittgen, 2001); healthy plants (CK) served as the control. Student’s *t*-test was used for statistical analyses (^∗^0.01 ≤ *P* < 0.05, ^∗∗^0.001 ≤ *P* < 0.01, ^∗∗∗^*P* < 0.001).

## Results

### Identification of a *F. oxysporum* f. sp. *lagenariae* Race

**FIGURE 1:**
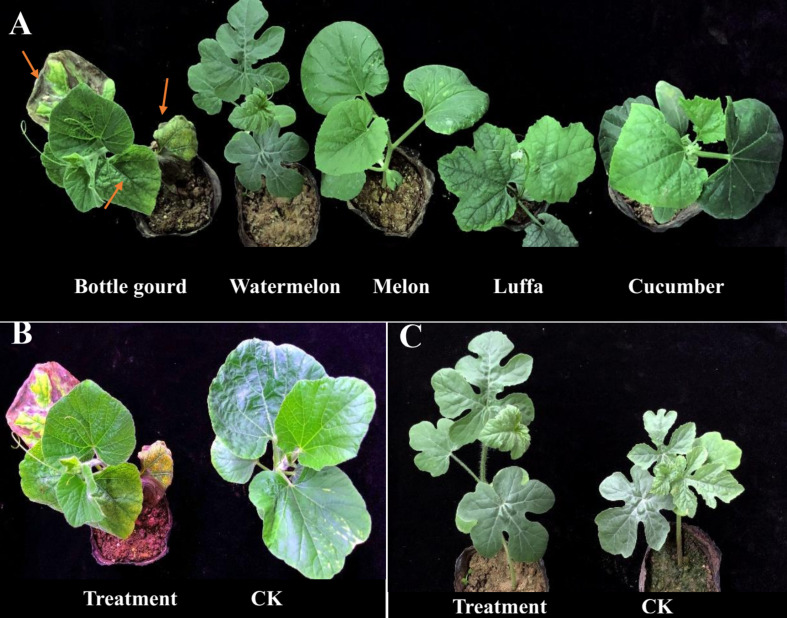
Morphology and *Fusarium* wilt (FW)-inoculated infestation response of cucurbit crops. **(A)** Bottle gourd was more susceptible than other crops after FW inoculation. **(B)** Plant phenotype of bottle gourd inoculated treatment group and control group after 10 days. **(C)** Plant phenotype of watermelon inoculated treatment group and control group after 10 days.

According to the conventional tissue separation method, purified strains from *Fusarium* wilt-infected plants were obtained. Through morphological identification of the colony, the microscopic view of its conidia, and PCR detection of its sequence (Supplementary Figure 1), we preliminarily identified the bottle gourd wilt isolates as *F. oxysporum* f. sp. Due to differences in the infectivity and pathogenicity of different strains to cucurbit crops, individual strains of *F. oxysporum* usually infect only one or few host species. Thus, to better distinguish the different races of *F. oxysporum* f. sp., we still relied on the special host for identification. The pathogenicity results showed that bottle gourd plants had obvious wilt infection symptoms, featured by the first and second leaves that were more than 50% wilted and the third and fourth leaves that were crumpled. However, there were no symptoms of wilt infection in watermelon, melon, cucumber, and luffa plants (Figure 1). Therefore, we proposed that the isolated *F. oxysporum* f. sp was a *F. oxysporum* f. sp. *lagenariae* race and was named physiological race ShaoX-1, which was used for the subsequent phenotypic identification of bottle gourd.

### Phenotypic Analysis of FW Resistance in the Natural Population

**FIGURE 2:**
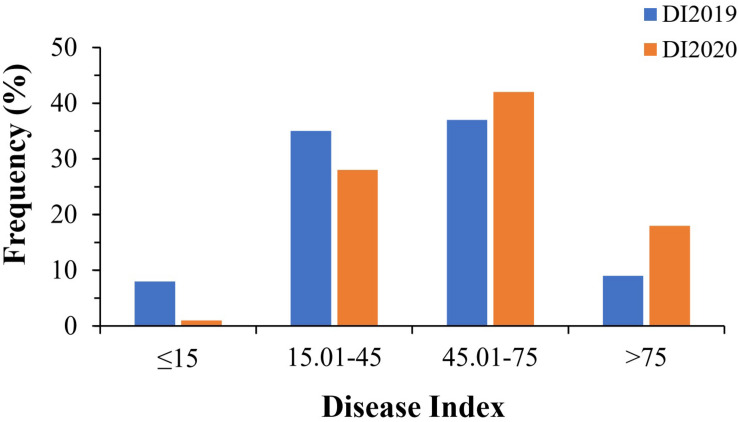
Phenotypic distribution of *Fusarium* wilt resistance in 89 bottle gourd accessions.

In this study, a total of 89 bottle gourd accessions were evaluated for resistance to *Fusarium* wilt in a glasshouse in 2019 (DI2019) and 2020 (DI2020), with two replicates per environment. DI, as an evaluation of FW resistance, had a wide range of phenotypic variation in the 2-year trials. The DI of all accessions ranged from 6 to 95%, with a mean value of 46% in 2019 (DI2019), and from 11 to 94%, with a mean value of 55% in 2020 (DI2020). The ANOVA results showed that the broad-sense heritability (*h*^2^) was 87.19% across the 2 years (Table 1), suggesting that the genetic effect played a predominant role in the phenotypic variation of FW resistance in bottle gourd. We divided the DI into five levels (Supplementary Figure 2): immune (level 0), highly resistant (level 1), resistant (level 2), susceptible (level 3), and highly susceptible (level 4), according to relevant previous studies (Gao et al., 2015; Xu et al., 2016). Only a tiny percentage of accessions had DI values less than 15% (8 in 2019 and 1 in 2020), whereas the majority of the accessions were within the range of 15.01–45% (35 in 2019 and 28 in 2020) and 45.01–75% (37 in 2019 and 42 in 2020). When DI exceeded 75%, there were 9 accessions in 2019 and 18 accessions in 2020 (Figure 2). Unfortunately, we did not select for any material that was immune to FW in the 2-year trials; only a small amount of material had high resistance to FW (Figure 2), which showed that the bottle gourd germplasm resource of FW resistance is scarce. The correlation coefficient between the 2-year trials was as high as 0.62 (Supplementary Figure 3), and the frequency distribution of DI was approximately normally distributed, which indicated that this natural population could be suitable for correlation analysis for FW resistance.

**TABLE 1:** Descriptive statistics and heritability (*h*^2^) of the *Fusarium* wilt disease index.

Trial	Maximum	Minimum	Mean	SD	CV (%)	Heritability (%)
DI2019	0.95	0.06	0.46	0.22	48.77	87.19
DI2020	0.94	0.11	0.55	0.22	41.21	
Mean	0.94	0.11	0.50	0.21	42.29	

*SD, standard deviation; CV (%), coefficient of variation.*

### SNP Marker Analysis

**FIGURE 3:**
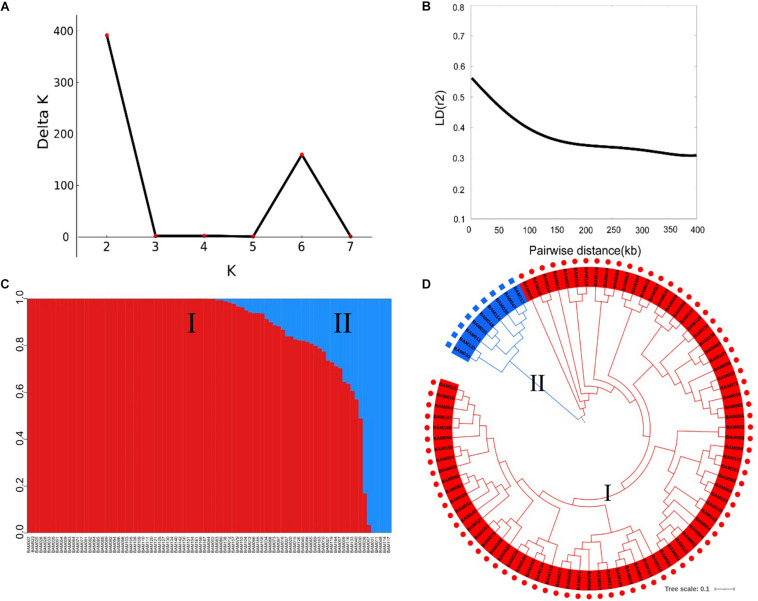
Population structure and linkage disequilibrium (LD) decay rate analysis of 89 bottle gourd accessions. **(A)** The mean delta *K* value, when *K* ranged from 2 to 8. **(B)** Decay of LD in the germplasm collection [from Xu et al., 2021, unpublished]. **(C)** Population structure of 89 bottle gourd accessions based on STRUCTURE software; subgroup1 is shown in red, and subgroup 2 is shown in blue. **(D)** Neighbor-joining tree of 89 bottle gourd accessions constructed by PHYLIP software.

The SNP markers used in this work resulted from RAD-sequencing by using the Illumina HiSeq platform. After removal of SNPs with a MAF of ≤0.01 and a heterozygous rate ≥25%, a total of 5,330 high-quality SNPs were retained for GWAS of the FW resistance trait. These SNPs covered all 11 chromosomes, with an uneven distribution across the genome (Table 2). The average density of SNP markers was about 59.37 kb/SNP. The maximum marker density was found on chromosome 11 (101.18 kb/SNP) followed by chromosome 6 (67.25 kb/SNP), whereas the minimum marker density was found on chromosome 1 (42.11 kb/SNP). Based on the SNP markers, we estimated the population structure of 89 bottle gourds using STRUCTURE software and cluster analysis. The delta *K* reached a sharp peak when *K* was 2. Therefore, this association population was divided into two subgroups, namely, subgroup 1 and subgroup 2 (Figures 3A,C). Subgroup 1 contained 80 accessions, and subgroup 2 was small and included nine accessions. A neighbor-joining result also classified the population into two subgroups, consistent with the population grouping result (Figure 3D). Because all accessions have some distant relationship, there were no primary factors, such as geographic distribution, affecting the population structure of the 89 accessions. Genotype data were subjected to correlation analysis of the free glutamate content trait in bottle gourd (Wu et al., 2017).

**TABLE 2:** Single-nucleotide polymorphism (SNP) marker distribution on 11 chromosomes of bottle gourd.

Chr.	Chromosome length (Mb)	Number of SNP	Density of SNP (kb/SNP)	PIC
chr1	28.39	674	42.11	0.10
chr2	29.76	631	47.17	0.14
chr3	30.34	563	53.90	0.13
chr4	32.30	637	50.70	0.11
chr5	35.14	553	63.55	0.12
chr6	26.83	399	67.25	0.12
chr7	23.92	389	61.49	0.13
chr8	23.22	505	45.98	0.10
chr9	19.99	370	54.03	0.12
chr10	26.30	400	65.75	0.10
chr11	21.15	209	101.18	0.12

To determine the mapping resolution of the GWAS, we estimated the genome-wide LD decay distance of the association population. The average LD decay distance was approximately 400 kb, where *r*^2^ = 0.3 for all chromosomes [half of its maximum value, from Xu et al., 2021, unpublished] (Figure 3B).

### Model Comparison for Correlation Analysis

To reduce a false positive association, we applied four kinds of association analysis models to GWAS for FW resistance in bottle gourd. Based on the mean value of DI across the 2 years, quantile–quantile (*Q*–*Q*) plots were drawn (Supplementary Figure 4). The results showed that there was a large deviation in the −log_10_*P* value between the observed values and the expected values in GLM (PCA) and GLM (*Q*) models, which indicated that the two models might cause a high false positive correlation. Due to the introduction of the covariable *K*, the observed −log_10_*P* values fit well with the expected values in the MLM (PCA + *K*) and MLM (*Q* + *K*) models, indicating that those two models could effectively control the false positive of the association analysis results. Taking into account the *Q*–*Q* plots of each environment, the MLM (*Q* + *K*) model (red scatter plot in Supplementary Figure 4) was selected for the follow-up association analysis for FW resistance.

### Genome-Wide Association Analysis

**FIGURE 4:**
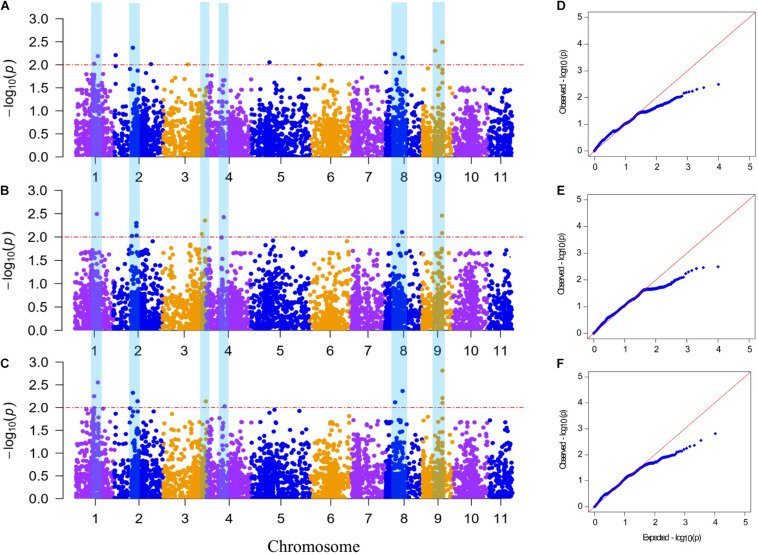
Manhattan plot and *Q*–*Q* plot of genome-wide association study for *Fusarium* wilt (FW) resistance in bottle gourd. **(A–C)** Manhattan plot for FW resistance in 2019 and 2020 and the mean across the 2 years, respectively, **(D–F)** *Q*–*Q* plot for FW resistance in 2019 and 2020 and the mean across the 2 years, respectively. The red horizontal dashed line indicates the genome-wide significance threshold (−log_10_*P* ≥ 2). The blue vertical bar spanning three graphs denotes 10 significantly correlated single-nucleotide polymorphism sites for FW resistance detected in at least two environments.

A GWAS was performed to detect SNPs associated with FW resistance between 5,330 SNP markers and 89 phenotype data points from the mean across the 2 years (aDI) and within an individual year (DI2019 and DI2020). The Manhattan plots and *Q*–*Q* plots for the GWAS results are shown in Figure 4. The GWAS result showed that 20 SNPs (with a significance threshold of *p* ≤ 0.01, −log_10_*P* ≥ 2.0) significantly associated with FW resistance were detected in at least one environment (Supplementary Table 3), including 12 SNPs from the 2019 data, 11 SNPs from the 2020 data, and 11 SNPs from the mean data. Among these SNPs, 10 significantly correlated SNP sites were detected in at least two environments, which were located on chromosomes 1, 2, 3, 4, 8, and 9, indicating that the FW resistance of bottle gourd is controlled by multiple genes (Figures 4A–C). The phenotypic variation explained by these sites ranged from 8.82 to 15.03% (Table 3). Among them, markers of BGReSe_14212 and BGReSe_14202 were located on chromosome 9, and those two SNP markers were within the range of the genome-wide LD block (400 kb). BGReSe_14202 was detected in all three environments with relatively high significant levels (−log_10_*P* = 2.81/2.49/2.46) and an effect on FW (*R*^2^ = 14.14%/13.90%/10.49%). Therefore, the region range of chromosome 9 may contain the major genes associated with FW resistance. On chromosome 8, two SNP markers were detected with a certain LD distance away. BGReSe_12911 was significantly correlated with FW resistance in all three environments, and BGReSe_12338 was detected in DI2019 and aDI. Two SNP markers, BGReSe_02569 and BGReSe_02108, were detected on chromosome 2. Of these two, BGReSe_02569 explained the largest phenotypic variation in DI2020 and aDI, *i*.*e*., 16.19 and 15.38%, respectively. BGReSe_02108 was detected in three environments, with a contribution rate for phenotypic variation of 12.60, 11.03, and 11.28%. BGReSe_01042 and BGReSe_00818 were located on chromosome 1. One of the markers, BGReSe_00818 (−log_10_*P* = 2.25/2.02), was significantly correlated with FW resistance in the two environments of DI2019 and aDI, and its contribution rate for phenotypic variation was 12.26 and 12.84%, respectively (Table 3).

**TABLE 3:** Significant markers associated with *Fusarium* wilt resistance in at least two environments.

Marker	Chromosome	Positive	Allelic	2019DI		2020DI		aDI	
				−log_10_*P*	*R*^2^ (%)	−log_10_*P*	*R*^2^ (%)	−log_10_*P*	*R*^2^ (%)
BGReSe_14212	9	13,476,930	A/G			2.10	9.76	2.08	8.82
BGReSe_14202	9	13,457,203	A/G	2.81	14.14	2.49	13.90	2.46	10.49
BGReSe_12911	8	11,449,774	A/G	2.36	10.14	2.16	10.31	2.10	10.33
BGReSe_12338	8	6,378,304	C/T	2.23	15.03			2.12	12.87
BGReSe_5941	4	12,071,538	C/T			2.42	13.60	2.03	9.88
BGReSe_5382	3	28,668,323	A/G			2.35	15.40	2.14	12.25
BGReSe_2569	2	15,601,788	A/G			2.14	16.19	2.03	15.38
BGReSe_2108	2	12,417,989	A/G	2.37	12.60	2.32	11.03	2.02	11.28
BGReSe_1042	1	14,684,871	C/T	2.55	11.06	2.49	12.83	2.19	12.30
BGReSe_818	1	12,140,445	C/T	2.25	12.26			2.02	12.84

### Prediction of Candidate Genes for FW Resistance

**FIGURE 5:**
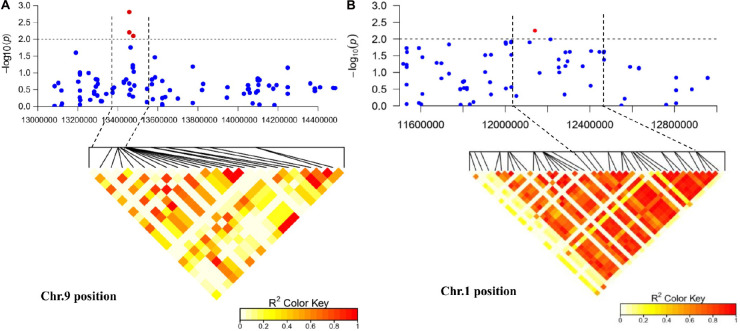
Regional Manhattan plot and linkage disequilibrium heat map of the candidate region of significantly associated single-nucleotide polymorphism (SNP) markers. **(A)** The candidate region of marker BGReSe_14212 on chromosome 9. **(B)** The candidate region of marker BGReSe_00818 on chromosome 1. The black horizontal dashed line indicates the genome-wide significance threshold. The region between the two black vertical dashed lines indicates the candidate region. Red pots indicate SNPs (−log_10_*P* ≥ 2.0) associated with *Fusarium* wilt resistance in at least one environment.

**FIGURE 6:**
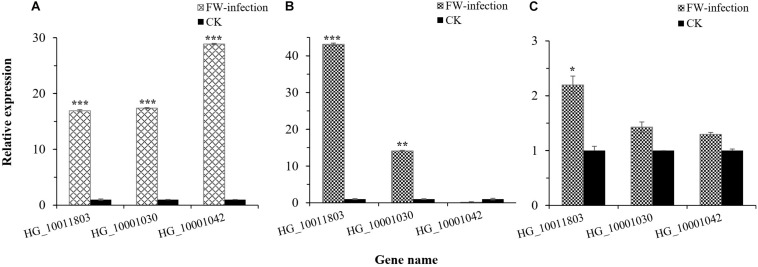
Relative expression level of three potential candidate genes in bottle gourd leaves. **(A)** Relative expression level of candidate genes in *Fusarium* wilt (FW)-infected and healthy (CK) YD-4. **(B)** Relative expression level of candidate genes in FW-infected and healthy (CK) Yin-10. **(C)** Relative expression level of candidate genes in FW-infected and healthy (CK) Hanbi. Statistical significance was detected by a two-tailed *t*-test (*0.01 ≤ *P* < 0.05, **0.001 ≤ *P* < 0.01, ***P < 0.001).

In this study, we were interested in the markers with the greatest effect, such as marker BGReSe_00818 (MAF = 1.16) on chromosome 1 and markers BGReSe_14202 (MAF = 1.09) and BGReSe_14212 (MAF = 1.07) on chromosome 9. To reduce the range of candidate regions, we performed LD block structure analysis. The results showed that BGReSe_14202 and BGReSe_14212 could form an obvious LD (±400 kb) block, meaning that these two SNPs were closely linked (Figure 5A). The candidate region of chromosome 9 was narrowed down to approximately 140 kb. This region contained 15 genes, of which two candidate genes were significantly associated with FW resistance of bottle gourd (Table 4). Both of them, *HG_GLEAN_10001030* (ethylene-responsive transcription factor RAP2) and *HG_GLEAN_10001042* (GDSL esterase), are involved in signaling pathways, such as resistance genes and hormone induction. LD block reduced the candidate region of BGReSe_00818 to about 415 kb, which contained seven genes (Figure 5B). Among them, *HG_GLEAN_10011803* encodes carboxylesterase and a CDPK-related kinase protein and plays a role in the signal transduction pathway. To confirm whether the potential candidate genes participated in the FW resistance pathway, the expression patterns of the three genes in both FW-infected and healthy bottle gourd plants were analyzed *via* qRT-PCR. The representative materials were selected from the association analysis population in this study. The DI of Yin-10 and Hanbi was 10.83 and 13.44%, respectively, and both were highly resistant (HR, level 1) to FW. The DI of YD-4 was 87.81%, i.e., highly susceptible (HS, level 4) to FW. The expression pattern of three potential candidate genes *HG_10011803*, *HG_10001030*, and *HG_10001042* in materials YD-4, Yin-10, and Hanbi is presented (Figure 6). Compared to healthy YD-4 (HS material, level 4), the expression levels of the three candidate genes were all significantly higher (*P* < 0.001) in the FW-infected group (3 days after infection) (Figure 6A). For Yin-10 and Hanbi (HR materials, level 1), the expression level of gene *HG_10011803* showed a significant difference (*P* < 0.05 and *P* < 0.001) between FW-infected and healthy groups. However, the expression levels of *HG_10001030* and *HG_10001042* in infected plants showed a higher or lower expression level than those in healthy Yin-10/Hanbi plants, without a significant difference (Figures 6B,C). Combining the above-mentioned interesting results, we speculated that *HG_10011803* is a major effect gene, whereas *HG_10001030* and *HG_10001042* might be the candidate genes involved in the FW resistance response in bottle gourd.

**TABLE 4:** Function annotation and genes in candidate intervals of *Fusarium* wilt resistance single-nucleotide polymorphisms.

Gene model	Chromosome	Start (bp)	End (bp)	Gene Ontology biological process descriptions
*HG_10001024*	9	13,370,905	13,396,585	Chloroplastic/mitochondrial isoform X1
*HG_10001026*	9	13,403,689	13,406,203	NA
*HG_10001028*	9	13,411,513	13,414,862	Mitochondrial dicarboxylate/tricarboxylate transporter
*HG_10001029*	9	13,422,565	13,425,196	Tubulin alpha-3 chain
HG_10001030*	9	13,426,782	13,427,351	Ethylene-responsive transcription factor
*HG_10001031*	9	13,435,260	13,440,011	Importin
*HG_10001032*	9	13,440,450	13,443,769	Importin-5
*HG_10001034*	9	13,447,396	13,453,363	Chloroplastic isoform X1
*HG_10001035*	9	13,453,903	13,460,368	Indole-3-acetaldehyde oxidase-like isoform X1
*HG_10001037*	9	13,467,477	13,475,348	Indole-3-acetaldehyde oxidase-like
*HG_10001040*	9	13,491,070	13,493,857	NADH–cytochrome b5 reductase 1-like isoform X3
*HG_10001041*	9	13,494,064	13,495,437	Chloroplastic-like isoform X1
HG_10001042*	9	13,495,614	13,500,201	GDSL esterase
*HG_10001043*	9	13,505,538	13,506,317	Protein YLS9-like
*HG_10001044*	9	13,510,514	13,520,956	La-related protein 1A
*HG_10011790*	1	12,064,283	12,065,568	Probable carboxylesterase 15
*HG_10011791*	1	12,073,500	12,084,249	Protein TRANSPARENT TESTA 12-like
*HG_10011796*	1	12,254,943	12,255,467	Hypothetical protein
*HG_10011797*	1	12,274,175	12,283,744	Hypothetical protein
*HG_10011798*	1	12,346,824	12,347,511	Hypothetical protein
*HG_10011799*	1	12,379,221	12,379,829	Zinc-finger homeodomain protein-like
HG_10011803*	1	12,423,049	12,429,778	CDPK-related kinase 1-like isoform X1

*potential candidate genes for FW resistance.

## Discussion

*Fusarium* wilt is one of the most important diseases throughout the world, which seriously affects the yield and quality of cucurbit crops (Miguel et al., 2004; Wechter et al., 2012; Oumouloud et al., 2013). The genetic mechanism of resistance to FW in cucurbit crops is complex, showing genetic diversity. However, there are no studies on the genetic effect and inheritance of genes governing FW resistance in bottle gourd, and molecular markers linked to FW resistance are also poorly reported.

Genome-wide association study has emerged as a powerful tool to study complex traits and genetic variations in SNP loci and has been successfully applied to different crops in recent years (Joobeur et al., 2004; Wang et al., 2009; Sabbavarapu et al., 2013; Zhang et al., 2014). Especially the general linear model and mixed linear model are still the common GWAS methods in plants (Huang et al., 2010; Li et al., 2013; Fang et al., 2017). GLM, based on a linear regression model, is usually used for the analysis of quantitative traits and discrete resistance traits. MLM, based on population structure (*Q*) and kinship (*K*) as covariance, could better reduce the false positive association (Yu et al., 2006). Taking into account the deviation between expected −log_10_*P* and observed −log_10_*P* in *Q*–*Q* plots, we finally selected the MLM (*Q* + *K*) (Supplementary Figure 4) as GWAS model for FW resistance. In this study, we used a GWAS to evaluate a population of 89 accessions for FW resistance under glasshouse inoculation conditions. A total of 20 SNP_*S*_ (*P* ≤ 0.01, −log_10_*P* ≥ 2.0)significantly associated with FW resistance were identified in at least one environment (Supplementary Table 3). These sites were distributed on seven chromosomes, which could explain the phenotypic variation up to 16.19%. Among them, 10 significantly correlated SNP sites were detected in at least two environments, which were located on chromosomes 1, 2, 3, 4, 8, and 9 (Figure 4). According to the reference genome sequence of “Hangzhou Gourd” (Wang et al., 2018), we preliminarily predicted three candidate genes in candidate regions or LD block regions of these 10 SNP markers (Figure 5). *HG_GLEAN_10011803*, a candidate gene, which was located 280 kb upstream of the BGReSe_00818 marker on Chr.1, encodes calcium-dependent protein kinase (CDPK) protein. There have been increasing studies confirming the involvement of CDPKs in plant disease resistance defense responses (Boudsocq and Sheen, 2013). For example, Loss-*AtCPK28* or overexpression-*AtCDPK1* mutants displayed enhanced responses to antibacterial immunity in *Arabidopsis* (Coca and Segundo, 2010; Monaghan et al., 2014). *SlCRK6* in tomato played a role in resistance to both *Sclerotinia sclerotiorum* and *Pseudomonas syringae* pv. *tomato* (*Pst*) DC3000 (Wang et al., 2016). *StCDPK5VK* in potato could increase resistance to late blight fungus through the production of ROS (Kobayashi et al., 2012). In addition, by conducting a qRT-PCR analysis, we found that the expression level of *HG_GLEAN_10011803* in FW-infected plants was significantly higher than that in healthy plants (Figure 6). Therefore, we inferred that the candidate gene *HG_GLEAN_10011803* might be related to the FW resistance of bottle gourd.

In the LD block region of another candidate marker BGReSe_14202, one candidate gene *HG_GLEAN_10001030*, located 50 kb upstream of this marker on chromosome 9, encoded the ethylene-responsive transcription factor (ERTF) RAP2 protein. ERTFs play an important regulatory role in plant signal transduction of disease resistance and stress resistance, and overexpression could improve plant disease resistance and stress resistance (Singh et al., 2002; Gutterson and Reuber, 2004). For example, *OsRAP2.6*-overexpressed plants showed improved resistance to rice blast fungus (Wamaitha et al., 2012). *TERF1* and *TSRF1* genes in tomato could be resistant to *Ralstonia solanacearum* and *Botrytis cinerea* (Huang et al., 2004; Zhang et al., 2004, 2008). Another candidate gene, *HG_10001042*, located 18 kb downstream of this marker on chromosome 9, is a member of the GDSL gene family. The GDSL gene family consists of a wide range of members and plays important roles in plant growth, development, and stress defense responses (Akoh et al., 2004; Chepyshko et al., 2012). Overexpressed GDSL genes, such as *AtGLIP1* and *CaGLIP1*, could enhance the resistance to a variety of pathogenic fungi (Hong et al., 2008; Lee et al., 2009; Naranjo et al., 2010). The qPR-PCR results showed that the expression levels of these two candidate genes were significantly increased in FW-infected YD-4 (HS material, level 4), while their expression levels were not significantly different before and after infection of Yin-10/Hanbi (HR materials, level 1) (Figure 6). Thus, we postulate that these three genes were candidate genes for FW resistance; in particular, *HG_GLEAN_10011803* might be a major effect gene. However, further evidence is needed to functionally validate this hypothesis. To our knowledge, this study is the first to perform GWAS for FW resistance in cucurbit crops. Our results provide the molecular tools for FW resistance selection and lay a foundation for candidate gene discovery. The resistant materials and SNP markers that we identified will promote breeding programs for FW-resistant bottle gourd.

## Data Availability Statement

The datasets presented in this study can be found in online repositories. The names of the repository/repositories and accession number(s) can be found in the article/Supplementary Material.

## Author Contributions

YL and GL conceived and designed the research. YL, YW, and JW performed the experiments. YL wrote the manuscript. YL, YW, and XinW constructed the population and collected phenotypes. XiaW, BW, and ZL carried out the field work. All authors analyzed the data and approved the submitted version.

## Conflict of Interest

The authors declare that the research was conducted in the absence of any commercial or financial relationships that could be construed as a potential conflict of interest.

## Footnotes

1 http://www.gourdbase.cn

2 http://www.gourdbase.cn

3 http://www.gourdbase.cn

4 https://www.idtdna.com/scitools/Applications/RealTimePCR/Default.aspx

## Supplementary Material

Supplementary Figure 1 Morphological features of wilted plant and *Fusarium oxysporum* f. sp. *lagenariae*. **(A)** Symptoms of wilted bottle gourd. **(B)** Symptoms of vascular bundle browning of wilted plants. **(C)** *F. oxysporum* colony of *Fusarium* wilt on potato dextrose agar. **(D)** Microscopic view of conidia of *F. oxysporum* f. sp. *lagenariae*.

Supplementary Figure 2 Plant symptoms of *Fusarium* wilt disease at levels 0–4 in bottle gourd. From left to right: level 0 (I), level 1 (HR), level 2 (R), level 3 (S), and level 4 (HS).

Supplementary Figure 3 Correlation analysis of disease index of 89 bottle gourd accessions between 2019 and 2020.

Supplementary Figure 4 *Q*–*Q* plot of genome-wide association study for *Fusarium* wilt resistance based on four different association analysis models. **(A)** Four different association analysis models of DI2019. **(B)** Four different association analysis models of DI2020. Different colors represent different models: blue, GLM (PCA); black, GLM (Q); green, MLM (PCA+K); red, MLM (Q+K).

Supplementary Table 1 Accession, origin, and disease index of 89 bottle gourd accessions used in this study.

Supplementary Table 2 Primer sequences used for qRT-PCR.

Supplementary Table 3 Significant markers associated with *Fusarium* wilt resistance in at least one environment.

## References

[B1] Akoh C. C. Lee G. C. Liaw Y. C. Huang T. H. Shaw J. F. (2004). GDSL family of serine esterases/lipases. *Prog. Lipid Res.* 43 534–552. 10.1016/j.plipres.2004.09.002 15522763

[B2] Anderson A. D. Weir B. S. (2007). A maximum-likelihood method for the estimation of pairwise relatedness in structured populations. *Genetics* 176 421–440. 10.1534/genetics.106.063149 17339212 PMC1893072

[B3] Atwell S. Huang Y. Vilhjálmsson B. Willems G. Horton M. Li Y. (2010). Genome-wide association study of 107 phenotypes in *Arabidopsis thaliana* inbred lines. *Nature* 465 627–631. 10.1038/nature08800 20336072 PMC3023908

[B4] Bhawna A. M. Arya L. Saha D. Sureja A. K. Pandey C. (2014). Population structure and genetic diversity in bottle gourd [*Lagenaria siceraria* (Mol.) Standl.] germplasm from India assessed by ISSR markers. *Plant Syst. Evol.* 300 767–773. 10.1007/s00606-014-1000-5

[B5] Bodah E. T. (2017). Root rot diseases in plants: a review of common causal agents and management strategies. *Agric. Res. Technol.* 5:555661. 10.19080/ARTOAJ.2017.05.555661

[B6] Boudsocq M. Sheen J. (2013). CDPKs in immune and stress signaling. *Trends Plant Sci.* 18 30–40. 10.1016/j.tplants.2012.08.008 22974587 PMC3534830

[B7] Catanzariti A. M. Lim G. T. Jones D. A. (2015). The tomato I-3 gene: a novel gene for resistance to *Fusarium* wilt disease. *New Phytol.* 207 106–118. 10.1111/nph.13348 25740416

[B8] Cha J. Y. Han S. Hong H. J. Cho H. Kim D. Kwon Y. (2016). Microbial and biochemical basis of a *Fusarium* wilt-suppressive soil. *ISME. J.* 10 119–129. 10.1038/ismej.2015.95 26057845 PMC4681868

[B9] Chepyshko H. Lai C. P. Huang L. M. Liu J. H. Shaw J. F. (2012). Multifunctionality and diversity of GDSL esterase/lipase gene family in rice (*Oryza sativa* L. japonica) genome: new insights from bioinformatics analysis. *BMC Genomics* 13:309. 10.1186/1471-2164-13-309 22793791 PMC3412167

[B10] Coca M. Segundo B. S. (2010). AtCPK1 calcium-dependent protein kinase mediates pathogen resistance in *Arabidopsis*. *Plant Mol. Biol.* 63 526–540. 10.1111/j.1365-313X.2010.04255.x 20497373

[B11] Ehlers J. D. Hall A. E. Patel P. N. Roberts P. A. Matthews W. C. (2000). Registration of ‘California blackeye 27’ cowpea. *Crop Sci.* 40 854–855. 10.2135/cropsci2000.403611x

[B12] Ehlers J. D. Sanden B. L. Frate C. A. Hall A. E. Roberts P. A. (2009). Registration of ‘California blackeye 50’ cowpea. *J. Plant Regist.* 3 236–240. 10.3198/jpr2009.01.0039crc

[B13] Erickson D. L. Smith B. D. Clarke A. C. Sandweiss D. H. Tuross N. (2005). An Asian origin for a 10,000-year-old domesticated plant in the Americas. *Proc. Natl. Acad. Sci. U.S.A.* 102 18315–18320. 10.1073/pnas.0509279102 16352716 PMC1311910

[B14] Evanno G. Regnaut S. Goudet J. (2005). Detecting the number of clusters of individuals using the software STRUCTURE: a simulation study. *Mol. Ecol.* 14 2611–2620. 10.1111/j.1365-294X.2005.02553.x 15969739

[B15] Fang C. Ma Y. M. Wu S. W. Liu Z. Wang Z. Yang R. (2017). Genome-wide association studies dissect the genetic networks underlying agronomical traits in soybean. *Genome Biol.* 18:161. 10.1186/s13059-017-1289-9 28838319 PMC5571659

[B16] Freeman S. Zveibil A. Vintal H. Maymon M. (2002). Isolation of nonpathogenic mutants of *Fusarium oxysporum* f. sp. melonis for biological control of *Fusarium* wilt in cucurbits. *Phytopathology* 92 164–168. 10.1094/phyto.2002.92.2.164 18943089

[B17] Gao P. Liu S. Zhu Q. L. Luan F. S. (2015). Marker-assisted selection of *Fusarium* wilt-resistant and gynoecious melon (*Cucumis melo* L.). *Genet. Mol. Res.* 14 16255–16264. 10.4238/2015 26662419

[B18] Gutterson N. Reuber T. L. (2004). Regulation of disease resistance pathways by AP2/ERF transcription factors. *Curr. Opin. Plant. Biol.* 7 465–471. 10.1016/j.pbi.2004.04.007 15231271

[B19] He J. Meng S. Zhao T. Xing G. Gai J. (2017). An innovative procedure of genome-wide association analysis fits studies on germplasm population and plant breeding. *Theor. Appl. Genet.* 130 2327–2343. 10.1007/s00122-017-2962-9 28828506

[B20] Heiser C. B. (1979). *Gourd Book.* Norman, OK: University of Oklahoma Press.

[B21] Hong J. K. Choi H. W. Hwang I. S. Kim D. S. Kim N. H. Choi D. S. (2008). Function of a novel GDSL-type pepper lipase gene, caglip1, in disease susceptibility and abiotic stress tolerance. *Planta* 227 539–558. 10.1007/s00425-007-0637-5 17929052

[B22] Huang X. H. Wei X. H. Sang T. Zhao Q. Feng Q. Zhao Y. (2010). Genome-wide association studies of 14 agronomic traits in rice landraces. *Nat. Genet.* 42 961–967. 10.1038/ng.695 20972439

[B23] Huang Z. Zhang Z. Zhang X. Zhang H. Huang D. Huang R. (2004). Tomato TERF1 modulates ethylene response and enhances osmotic stress tolerance by activating expression of downstream genes. *FEBS. Lett.* 573 110–116. 10.1016/j.febslet.2004.07.064 15327984

[B24] Joobeur T. King J. J. Nolin S. J. Thomas C. E. Dean R. A. (2004). The *Fusarium* wilt resistance locus Fom-2 of melon contains a single resistance gene with complex features. *Plant J.* 39 283–297. 10.1111/j.1365-313X.2004.02134.x 15255859

[B25] Kang H. M. Zaitlen N. A. Wade C. M. Kirby A. Eskin E. (2008). Efficient control of population structure in model organism association mapping. *Genetics* 178 1709–1723. 10.1534/genetics.107.080101 18385116 PMC2278096

[B26] Katan T. (1994). Physiologic races and vegetative compatibility groups of *Fusarium oxysporum* f. sp. melonis in Israel. *Phytopathology* 84 153–157. 10.1094/Phyto-84-153

[B27] Khan N. Maymon M. Hirsch A. M. (2017). Combating *Fusarium* infection using *Bacillus*-based antimicrobials. *Microorganisms* 5:75. 10.3390/microorganisms5040075 29165349 PMC5748584

[B28] Kobayashi M. Yoshioka M. Asai S. Nomura H. Kuchimura K. Mori H. (2012). StCDPK5 confers resistance to late blight pathogen but increases susceptibility to early blight pathogen in potato via reactive oxygen species burst. *New Phytol.* 196 223–237. 10.1111/j.1469-8137.2012.04226.x 22783903

[B29] Lambel S. Lanini B. Vivoda E. Fauve J. Patrick Wechter W. Harris-Shultz K. R. (2014). A major QTL associated with *Fusarium oxysporum* race 1 resistance identified in genetic populations derived from closely related watermelon lines using selective genotyping and genotyping-by-sequencing for SNP discovery. *Theor. Appl. Genet.* 127 2105–2115. 10.1007/s00122-014-2363-2 25104326

[B30] Lee D. S. Kim B. K. Kwon S. J. Jin H. C. Park O. K. (2009). *Arabidopsis* GDSL lipase 2 plays a role in pathogen defense via negative regulation of auxin signaling. *Biochem. Biophys. Res. Commun.* 379 1038–1042. 10.1016/j.bbrc.2009.01.006 19146828

[B31] Lee J. M. (1994). Cultivation of grafted vegetables I. current status, grafting methods, and benefits. *HortScience* 29 235–239. 10.21273/hortsci.29.4.235

[B32] Li H. Peng Z. Yang X. Wang W. Fu J. Wang J. (2013). Genome-wide association study dissects the genetic architecture of oil biosynthesis in maize kernels. *Nat. Genet.* 45 43–50. 10.1038/ng.2484 23242369

[B33] Li T. Ma X. Li N. Zhou L. Liu Z. Han H. (2017). Genome-wide association study discovered candidate genes of Verticillium wilt resistance in upland cotton (*Gossypium hirsutum* L.). *Plant Biotechnol. J.* 15 1520–1532. 10.1111/pbi.12734 28371164 PMC5698051

[B34] Li Y. Reif J. C. Ma Y. Hong H. Liu Z. Chang R. (2015). Targeted association mapping demonstrating the complex molecular genetics of fatty acid formation in soybean. *BMC Genomics* 16:841. 10.1186/s12864-015-2049-4 26494482 PMC4619020

[B35] Livak K. J. Schmittgen T. D. (2001). Analysis of relative gene expression data using real-time quantitative PCR and the 2−ΔΔCT method. *Methods* 25 402–408. 10.1006/meth.2001.1262 11846609

[B36] Martyn R. D. Bruton B. D. (1989). An initial survey of the United States for races of *Fusarium oxysporum* f. sp. *niveum*. *HortScience* 24 696–698.

[B37] Miguel A. Maroto J. V. Bautista A. S. Baixauli C. Cebolla V. Pascual B. (2004). The grafting of triploid watermelon is an advantageous alternative to soil fumigation by methyl bromide for control of *Fusarium* wilt. *Sci. Hortic.* 103 9–17. 10.1016/j.scienta.2004.04.007

[B38] Monaghan J. Matschi S. Shorinola O. Rovenich H. Matei A. Segonzac C. (2014). The calcium-dependent protein kinase CPK28 buffers plant immunity and regulates BIK1 turnover. *Cell Host Microbe* 16 605–615. 10.1016/j.chom.2014.10.007 25525792

[B39] Morimoto Y. Mvere B. (2004). “Lagenaria siceraria,” in *Plant Resources of Tropical Africa 2*, eds Grubben G. J. H. Denton O. A. (Wageningen: Backhuys Publishers), 353–358.

[B40] Naranjo M. A. Forment J. Roldán M. Serrano R. Vicente O. (2010). Overexpression of *Arabidopsis thaliana* LTL1, a salt-induced gene encoding a GDSL-motif lipase, increases salt tolerance in yeast and transgenic plants. *Plant Cell Environ.* 29 1890–1900. 10.1111/j.1365-3040.2006.01565.x 16930315

[B41] Oumouloud A. El-Otmani M. Chikh-Rouhou H. Garcés Claver A. González Torres R. Perl-Treves R. (2013). Breeding melon for resistance to *Fusarium* wilt: recent developments. *Euphytica* 192 155–169. 10.1007/s10681-013-0904-4

[B42] Pottorff M. Li G. Ehlers J. D. Close T. J. Roberts P. A. (2013). Genetic mapping, synteny, and physical location of two loci for *Fusarium oxysporum* f. sp. *tracheiphilum* race 4 resistance in cowpea [*Vigna unguiculata* (L.) Walp]. *Mol. Breed.* 33 779–791. 10.1007/s11032-013-9991-0 24659904 PMC3956937

[B43] Pottorff M. Wanamaker S. Ma Y. Q. Ehlers J. D. Roberts P. A. Close T. J. (2012). Genetic and physical mapping of candidate genes for resistance to *Fusarium oxysporum* f.sp. *tracheiphilum* race 3 in cowpea [*Vigna unguiculata* (L.) Walp]. *PLoS One* 7:e41600. 10.1371/journal.pone.0041600 22860000 PMC3409238

[B44] Purcell S. Neale B. Todd-Brown K. Thomas L. Ferreira M. A. Bender D. (2007). PLINK: a tool set for whole-genome association and population-based linkage analyses. *Am. J. Hum. Genet.* 81 559–575. 10.1086/519795 17701901 PMC1950838

[B45] Sabbavarapu M. M. Sharma M. Chamarthi S. K. Swapna N. Rathore A. Thudi M. (2013). Molecular mapping of QTLs for resistance to *Fusarium* wilt (race 1) and *Ascochyta* blight in chickpea (*Cicer arietinum* L.). *Euphytica* 193 121–133. 10.1007/s10681-013-0959-2

[B46] Shen Z. Li X. X. (2008). *Descriptions and Data Standard for Bottle Gourd [Lagenaria siceraria (Mol.) Standl.].* Beijing: China Agriculture Press, 56–58.

[B47] Simons G. Groenendijk J. Wijbrandi J. Reijans M. Groenen J. Diergaarde P. (1998). Dissection of the *Fusarium* I2 gene cluster in tomato reveals six homologs and one active gene copy. *Plant Cell* 10 1055–1068. 10.1105/tpc.10.6.1055 9634592 PMC144031

[B48] Singh K. B. Foley R. C. Luis O. S. (2002). Transcription factors in plant defense and stress responses. *Curr. Opin. Plant Biol.* 5 430–436. 10.1016/S1369-5266(02)00289-3 12183182

[B49] Singh V. K. Singh H. B. Upadhyay R. S. (2017). Role of fusaric acid in the development of ‘*Fusarium* wilt’ symptoms in tomato: physiological, biochemical and proteomic perspectives. *Plant Physiol. Biochem.* 118 320–332. 10.1016/j.plaphy.2017.06.028 28683401

[B50] Wamaitha M. J. Yamamoto R. Wong H. L. Kawasaki T. Kawano Y. Shimamoto K. (2012). OsRap2.6 transcription factor contributes to rice innate immunity through its interaction with receptor for activated kinase-C1 (RACK1). *Rice* 5:35. 10.1186/1939-8433-5-35 24280008 PMC4883712

[B51] Wang J. P. Xu Y. P. Munyampundu J. P. Liu T. Y. Cai X. Z. (2016). Calcium-dependent protein kinase (CDPK) and CDPK-related kinase (CRK) gene families in tomato: genome-wide identification and functional analyses in disease resistance. *Mol. Genet. Genomics* 291 661–676. 10.1007/s00438-015-1137-0 26520101

[B52] Wang P. Su L. Qin L. Hu B. Guo W. Zhang T. (2009). Identification and molecular mapping of a *Fusarium* wilt resistant gene in upland cotton. *Theor. Appl. Genet.* 119 733–739. 10.1007/s00122-009-1084-4 19506830

[B53] Wang Y. Xu P. Wu X. H. Wu X. Y. Wang B. G. Huang Y. P. (2018). GourdBase: a genome-centered multi-omics database for the bottle gourd (*Lagenaria siceraria*), an economically important cucurbit crop. *Sci. Rep.* 8:3604. 10.1038/s41598-018-22007-3 29483591 PMC5827520

[B54] Wechter W. P. Kousik C. Mcmillan M. Leviet A. (2012). Identification of resistance to *Fusarium oxysporum* f. sp *niveum* race 2 in *Citrullus lanatus* var. *citroides* plant introductions. *HortScience* 47 334–338. 10.21273/HORTSCI.47.3.334

[B55] Whitaker T. W. (1971). *Endemism and Pre-Columbian Migration of Bottle Gourd, Lagenaria siceraria (Mol.) Standl.* Austin, TX: Man across the Sea, University of Press, 64–69.

[B56] Wu X. Xu P. Wang B. Lu Z. Li G. (2017). Genome-wide association analysis of free glutamate content, a key factor conferring umami taste in the bottle gourd [*Lagenaria siceraria* (Mol.) standl.]. *Sci. Horticult.* 225 795–801. 10.1016/j.scienta.2017.08.015

[B57] Xu P. Wu X. Luo J. Wang B. Liu Y. Ehlers J. D. (2011). Partial sequencing of the bottle gourd genome reveals markers useful for phylogenetic analysis and breeding. *BMC Genomics* 12:467. 10.1186/1471-2164-12-467 21942996 PMC3188536

[B58] Xu P. Xu S. Wu X. Tao Y. Wang B. Wang S. (2014). Population genomic analyses from low-coverage RAD-Seq data: a case study on the non-model cucurbit bottle gourd. *Plant J.* 77 430–442. 10.1111/tpj.12370 24320550

[B59] Xu X. W. Yu T. Xu R. Shi Y. Lin X. Xu Q. (2016). Fine mapping of a dominantly inherited powdery mildew resistance major-effect QTL, Pm1.1, in cucumber identifies a 41.1kb region containing two tandemly arrayed cysteine-rich receptor-like protein kinase genes. *Theor. Appl. Genet.* 129 507–516. 10.1007/s00122-015-2644-4 26660669

[B60] Yang N. Lu Y. Yang X. H. Huang J. Zhou Y. Ali F. H. (2014). Genome wide association studies using a new nonparametric model reveal the genetic architecture of 17 agronomic traits in an enlarged maize association panel. *PLoS Genet.* 10:e1004573. 10.1371/journal.pgen.1004573 25211220 PMC4161304

[B61] Yetisir H. Sari N. (2003). Effect of different rootstock on plant growth, yield and quality of watermelon. *Aust. J. Exp. Agric.* 43 1269–1274. 10.1071/EA02095

[B62] Yu J. Pressoir G. Briggs W. H. Vroh B. I. Yamasaki M. Doebley J. F. (2006). A unified mixed-model method for association mapping that accounts for multiple levels of relatedness. *Nat. Genet.* 38 203–208. 10.1038/ng1702 16380716

[B63] Zhang H. Yang Y. Zhang Z. Chen J. Wang X. C. Huang R. (2008). Expression of the ethylene response factor gene TSRF1 enhances abscisic acid responses during seedling development in tobacco. *Planta* 228 777–787. 10.1007/s00425-008-0779-0 18597110

[B64] Zhang H. Zhang D. Chen J. Yang Y. Huang Z. Huang D. (2004). Tomato stress-responsive factor TSRF1 interacts with ethylene responsive element GCC box and regulates pathogen resistance to *Ralstonia solanacearum*. *Plant Mol. Biol.* 55 825–834. 10.1007/s11103-004-2140-8 15604719

[B65] Zhang S. P. Miao H. Yang Y. H. Xie B. Y. Wang Y. Gu X. F. (2014). A major quantitative trait locus conferring resistance to *Fusarium* wilt was detected in cucumber by using recombinant inbred lines. *Mol. Breed.* 34 1805–1815. 10.1007/s11032-014-0140-1

[B66] Zhang X. Zhao J. Bu Y. Xue D. Liu Z. Li X. (2018). Genome-wide association studies of soybean seed hardness in the Chinese mini core collection. *Plant Mol. Biol. Rep.* 36 605–617. 10.1007/s11105-018-1102-2

[B67] Zhang Z. Ersoz E. Lai C. Q. Todhunter R. J. Tiwari H. K. Gore M. A. (2010). Mixed linear model approach adapted for genome-wide association studies. *Nat. Genet.* 42 355–360. 10.1038/ng.546 20208535 PMC2931336

[B68] Zhao Y. Wang H. Chen W. Li Y. (2014). Genetic structure, linkage disequilibrium and association mapping of Verticillium wilt resistance in elite cotton (*Gossypium hirsutum* L.) germplasm population. *PLoS One* 9:e86308. 10.1371/journal.pone.0086308 24466016 PMC3900507

[B69] Zink F. W. Gubler W. D. (1985). Inheritance of resistance in muskmelon to *Fusarium* wilt. *J. Am. Soc. Hort.* 110 600–604.

