# Supplementary figures and images for: Novel Genomic Regions of Fusarium Wilt Resistance in Bottle Gourd [Lagenaria siceraria (Mol.) Standl.] Discovered in Genome-Wide Association Study
Source: Front Plant Sci. 2021 May 7;12:650157. doi: 10.3389/fpls.2021.650157 (PMC8137845; doi:10.3389/fpls.2021.650157)

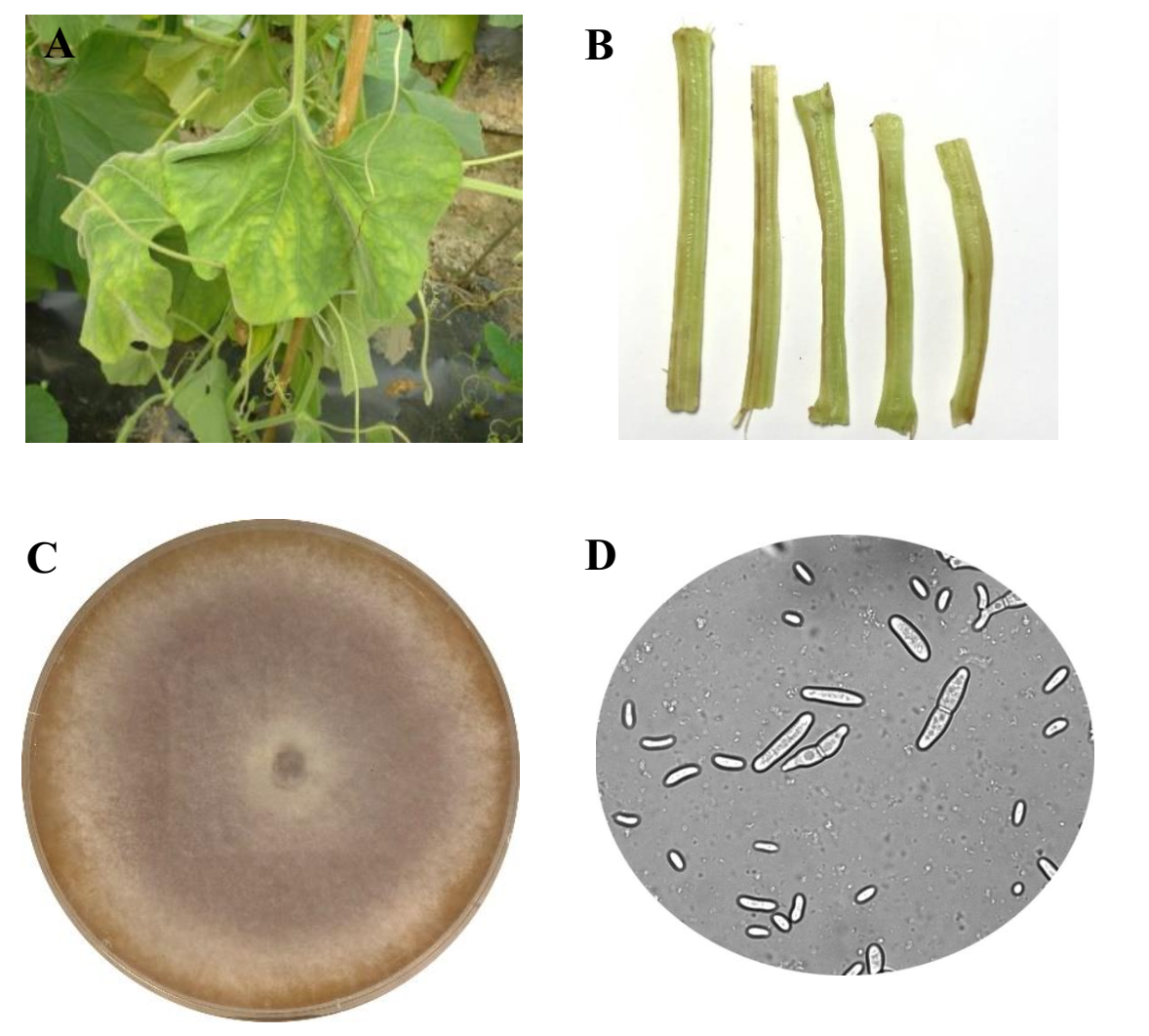

Supplement: Supplementary Figure 1 — Morphological features of wilted plant and Fusarium oxysporum f. sp. lagenariae. (A) Symptoms of wilted bottle gourd. (B) Symptoms of vascular bundle browning of wilted plants. (C) F. oxysporum colony of Fusarium wilt on potato dextrose agar. (D) Microscopic view of conidia of F. oxysporum f. sp. lagenariae. [file Image_1.JPEG]

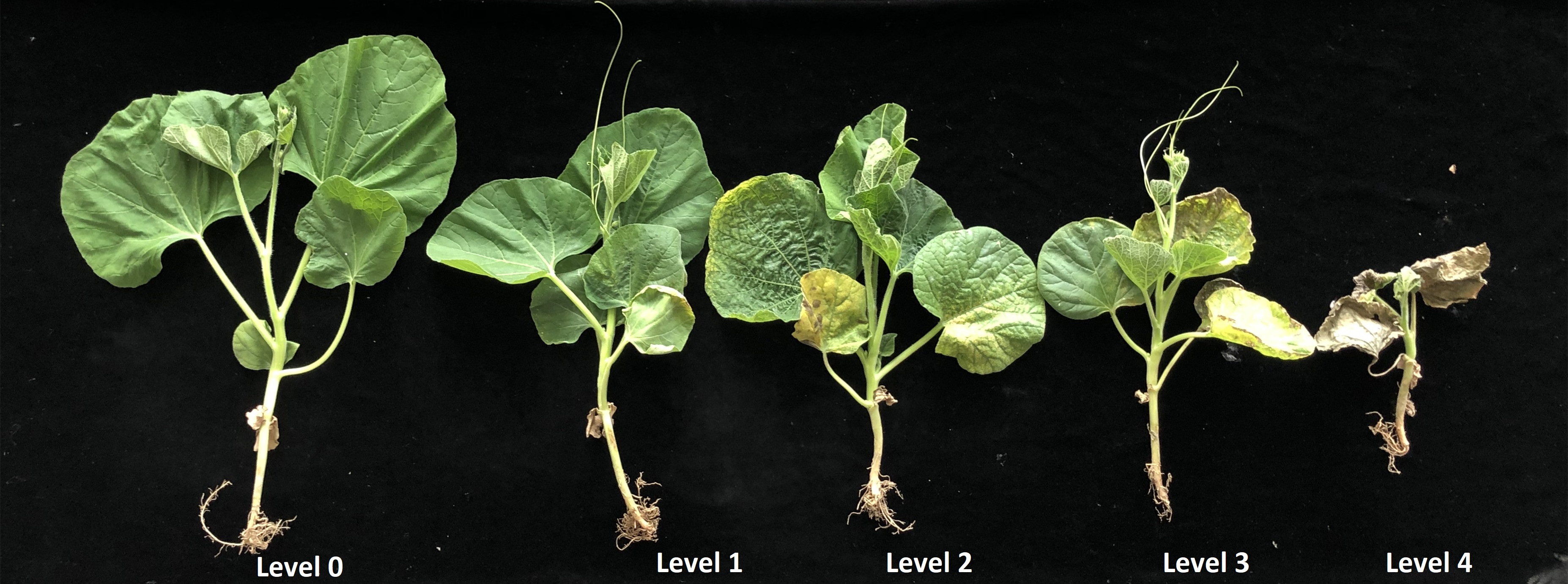

Supplement: Supplementary Figure 2 — Plant symptoms of Fusarium wilt disease at levels 0–4 in bottle gourd. From left to right: level 0 (I), level 1 (HR), level 2 (R), level 3 (S), and level 4 (HS). [file Image_2.JPEG]

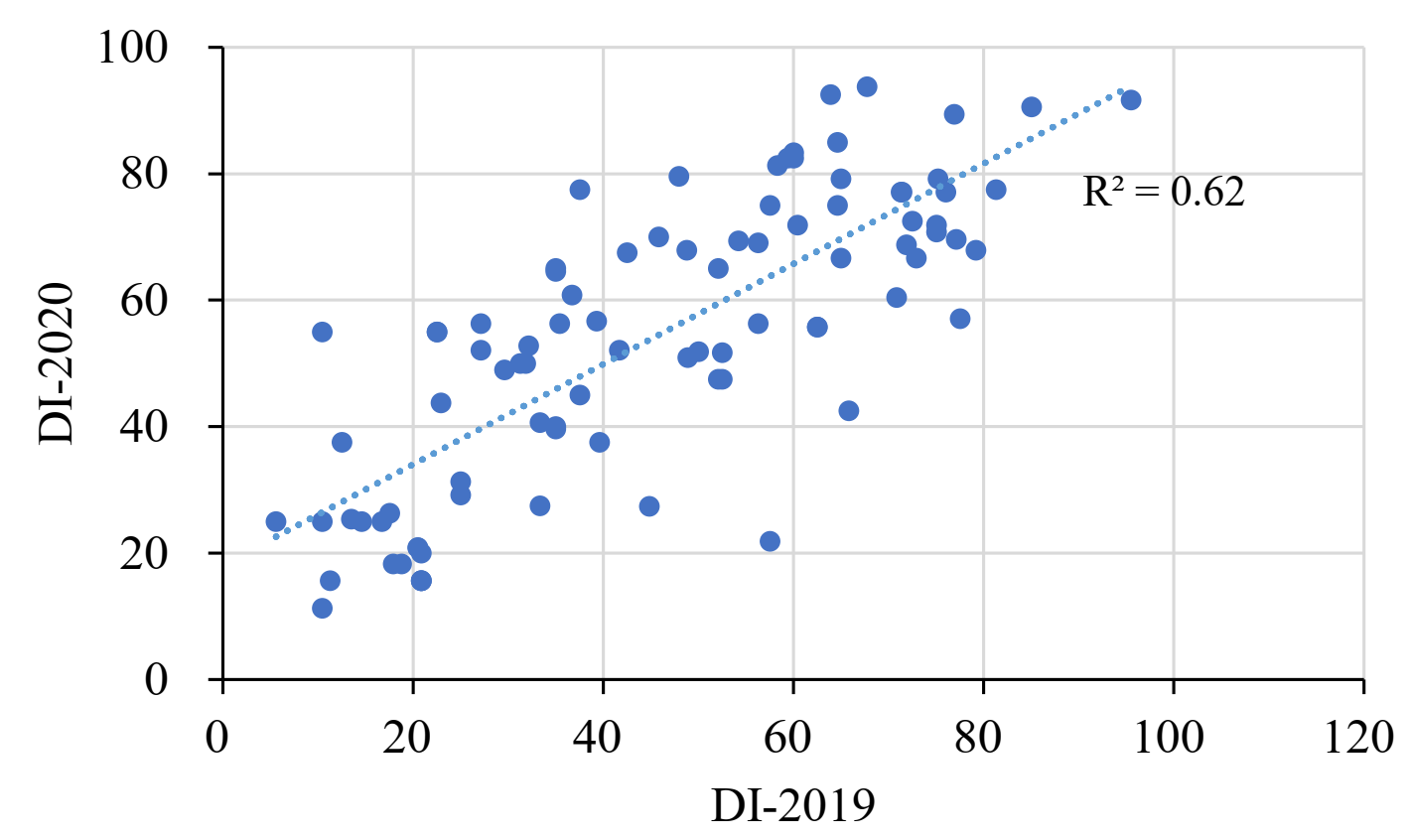

Supplement: Supplementary Figure 3 — Correlation analysis of disease index of 89 bottle gourd accessions between 2019 and 2020. [file Image_3.JPEG]

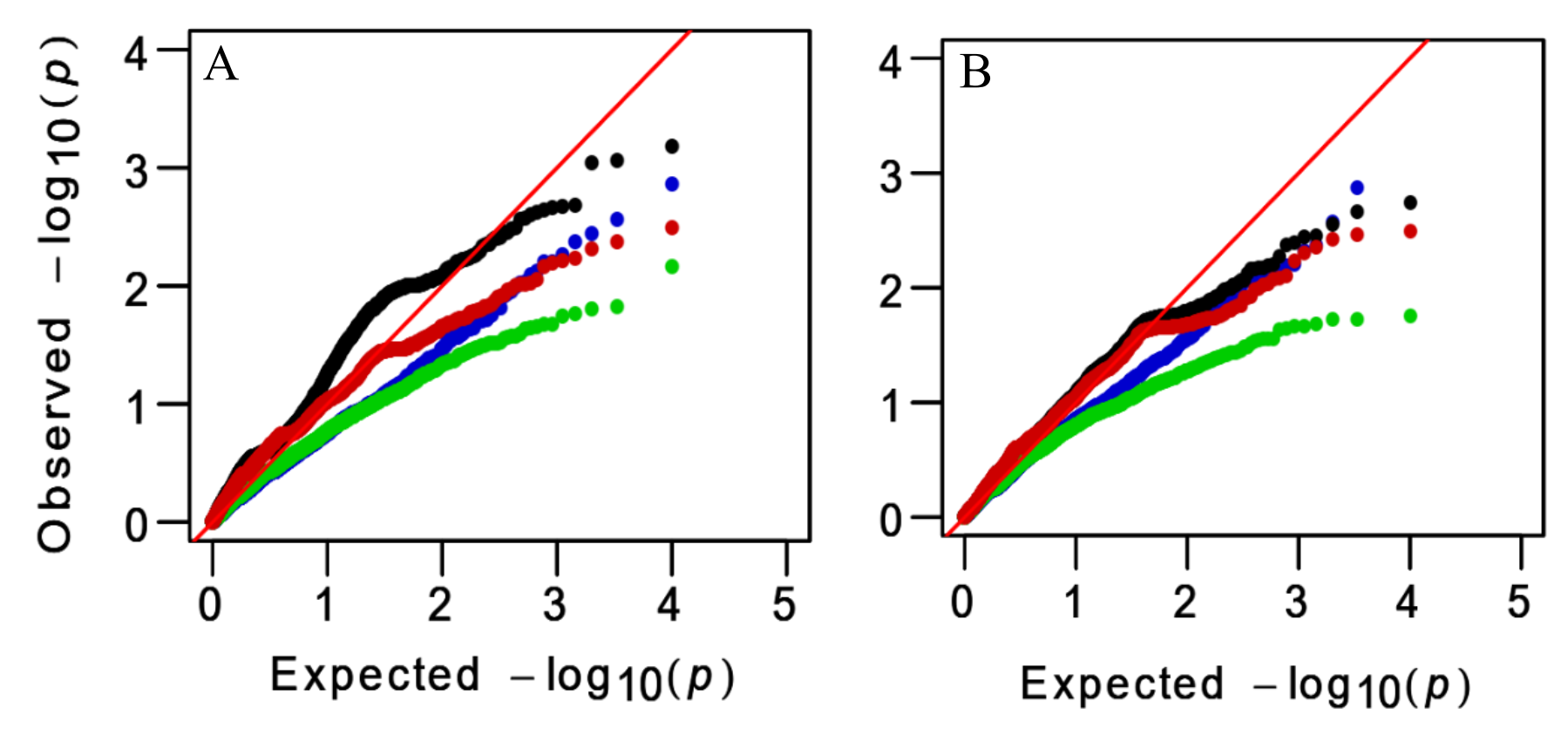

Supplement: Supplementary Figure 4 — Q–Q plot of genome-wide association study for Fusarium wilt resistance based on four different association analysis models. (A) Four different association analysis models of DI2019. (B) Four different association analysis models of DI2020. Different colors represent different models: blue, GLM (PCA); black, GLM (Q); green, MLM (PCA+K); red, MLM (Q+K). [file Image_4.JPEG]
