# Supplementary material for: Novel Genomic Regions of Fusarium Wilt Resistance in Bottle Gourd [Lagenaria siceraria (Mol.) Standl.] Discovered in Genome-Wide Association Study
Source: Front Plant Sci. 2021 May 7;12:650157. doi: 10.3389/fpls.2021.650157 (PMC8137845; doi:10.3389/fpls.2021.650157)
Supplement: Supplementary Table 1 — Accession, origin, and disease index of 89 bottle gourd accessions used in this study. [file Data_Sheet_1.docx]

**Supplementary Table 1.** Accession, origin and disease index (DI) of 89 bottle gourd accessions used in this study

| Accession ID | Accession name | Collection site | DI2019 | DI2020 |
| --- | --- | --- | --- | --- |
| BAM002 | J104 | Hunan province  (Central China) | 0.650 | 0.792 |
| BAM007 | J051 | Fujian province  (East China) | 0.146 | 0.250 |
| BAM009 | Zhenmu | Shandong province  (East China) | 0.318 | 0.500 |
| BAM010 | J022 | Fujian province  (East China) | 0.175 | 0.263 |
| BAM011 | PS30 | Former Serbia and Monteneg | 0.955 | 0.917 |
| BAM012 | I87 | Guizhou province  (Southwest China) | 0.125 | 0.375 |
| BAM013 | Hulupu | Zhejiang province  (East China) | 0.205 | 0.208 |
| BAM015 | I82 | Guizhou province  (Southwest China) | 0.425 | 0.675 |
| BAM016 | JH-2 | Zhejiang province  (East China) | 0.750 | 0.719 |
| BAM020 | Ganxin | Jiangxi province  (East China) | 0.521 | 0.475 |
| BAM022 | I214 | Hubei province  (Central China) | 0.729 | 0.667 |
| BAM025 | YD-5 | Guangdong province  (Southern China) | 0.250 | 0.313 |
| BAM026 | YD-9 | Guangdong province  (Southern China) | 0.167 | 0.250 |
| BAM027 | JH-8 | Zhejiang province  (East China) | 0.271 | 0.521 |
| BAM028 | YD-4 | Guangdong province  (Southern China) | 0.850 | 0.906 |
| BAM030 | YD-1 | Guangdong province  (Southern China) | 0.208 | 0.156 |
| BAM031 | Yunnan | Yunnan province  (Southwest China) | 0.350 | 0.400 |
| BAM033 | Yuepu | Zhejiang province  (East China) | 0.354 | 0.563 |
| BAM034 | Nanqingnan | Zhejiang province  (East China) | 0.396 | 0.375 |
| BAM035 | J010 | Sichuan province  (Southwest China) | 0.321 | 0.579 |
| BAM038 | Yuanhulu | Zhejiang province  (East China) | 0.750 | 0.708 |
| BAM040 | Dongyang 5 | Zhejiang province  (East China) | 0.208 | 0.156 |
| BAM044 | Jinhua | Zhejiang province  (East China) | 0.333 | 0.406 |
| BAM045 | IY-4 | Zhejiang province  (East China) | 0.375 | 0.450 |
| BAM047 | Tounianpu | Zhejiang province  (East China) | 0.658 | 0.425 |
| BAM048 | PS79 | Mexico | 0.179 | 0.183 |
| BAM051 | G26 | Guizhou province  (Southwest China) | 0.350 | 0.650 |
| BAM052 | G5dongyang | Zhejiang province  (East China) | 0.104 | 0.250 |
| BAM054 | J098BC | Zhejiang province  (East China) | 0.296 | 0.490 |
| BAM057 | J120 | Jiangsu province  (East China) | 0.752 | 0.792 |
| BAM059 | J138 | Jiangxi province  (East China) | 0.771 | 0.696 |
| BAM063 | G6 | Zhejiang province  (East China) | 0.775 | 0.571 |
| BAM064 | NXZ | Hubei province  (Central China) | 0.646 | 0.85 |
| BAM065 | YW | Zhejiang province  (East China) | 0.479 | 0.796 |
| BAM066 | I182 | Sichuan province  (Southwest China) | 0.500 | 0.677 |
| BAM068 | I6 | Gansu province  (Northwest China) | 0.542 | 0.694 |
| BAM069 | I32 | Fujian province  (East China) | 0.367 | 0.608 |
| BAM071 | J063 | Gansu province  (Northwest China) | 0.500 | 0.519 |
| BAM073 | Duanguang137 | Guangdong province  (Southern China) | 0.575 | 0.219 |
| BAM077 | I180 | Sichuan province  (Southwest China) | 0.563 | 0.691 |
| BAM079 | Fujian | Fujian province  (East China) | 0.525 | 0.475 |
| BAM080 | Jiushanyuan gourd | Zhejiang province  (East China) | 0.313 | 0.500 |
| BAM081 | G17 | Anhui province  (East China) | 0.604 | 0.719 |
| BAM082 | Changhu | unclear | 0.575 | 0.275 |
| BAM083 | LS | Zhejiang province  (East China) | 0.250 | 0.292 |
| BAM084 | Quanhua | Zhejiang province  (East China) | 0.813 | 0.775 |
| BAM085 | J108 | Hunan province  (Central China) | 0.600 | 0.833 |
| BAM086 | I77 | Guangdong province  (Southern China) | 0.708 | 0.604 |
| BAM088 | I103 | Hunan province  (Central China) | 0.350 | 0.396 |
| BAM089 | I16 | Fujian province  (East China) | 0.375 | 0.775 |
| BAM092 | Qingyu | unclear | 0.719 | 0.688 |
| BAM094 | ZH | Jiangxi province  (East China) | 0.229 | 0.438 |
| BAM096 | G14 | Tianjin province  (North China) | 0.725 | 0.725 |
| BAM097 | J089 | Guizhou province  (Southwest China) | 0.350 | 0.646 |
| BAM098 | He1 | unclear | 0.646 | 0.750 |
| BAM103 | J6 | Zhejiang province  (East China) | 0.639 | 0.925 |
| BAM104 | J125 | Jiangsu province  (East China) | 0.333 | 0.275 |
| BAM105 | Yuanhu No.1 | Zhejiang province  (East China) | 0.769 | 0.894 |
| BAM108 | Qingxiu | Hubei province  (Central China) | 0.271 | 0.563 |
| BAM109 | G13 | Hubei province  (Central China) | 0.488 | 0.679 |
| BAM110 | G15 | Jiangsu province  (East China) | 0.583 | 0.813 |
| BAM111 | G5 | Zhejiang province  (East China) | 0.104 | 0.550 |
| BAM114 | Bianpu | Taiwan province  (East China) | 0.188 | 0.183 |
| BAM116 | Yongzhen | Zhejiang province  (East China) | 0.208 | 0.200 |
| BAM117 | ZS | Zhejiang province  (East China) | 0.417 | 0.521 |
| BAM119 | I85 | Guangdong province  (Southern China) | 0.056 | 0.250 |
| BAM120 | YD-3 | Guangdong province  (Southern China) | 0.225 | 0.550 |
| BAM121 | Qingxiang | unclear | 0.393 | 0.567 |
| BAM125 | G11-2 | Zhejiang province  (East China) | 0.563 | 0.563 |
| BAM126 | Qingpiyuanpu | Zhejiang province  (East China) | 0.208 | 0.156 |
| BAM127 | Hanbi | Hubei province  (Central China) | 0.113 | 0.156 |
| BAM130 | YD-7 | Guangdong province  (Southern China) | 0.713 | 0.771 |
| BAM133 | YD-8 | Guangdong province  (Southern China) | 0.205 | 0.208 |
| BAM134 | J099 | Henan province  (Central China) | 0.650 | 0.667 |
| BAM138 | J106 | Hunan province  (Central China) | 0.458 | 0.700 |
| BAM140 | J137 | Jiangxi province  (East China) | 0.225 | 0.550 |
| BAM142 | G8 | Zhejiang province  (East China) | 0.521 | 0.650 |
| BAM150 | I62 | Anhui province  (East China) | 0.625 | 0.557 |
| BAM151 | Nanxiu | Hubei province  (Central China) | 0.135 | 0.254 |
| BAM153 | Shangyuduanpu | Zhejiang province  (East China) | 0.677 | 0.938 |
| BAM154 | Yaohulu | Zhejiang province  (East China) | 0.760 | 0.771 |
| BAM157 | G6 | Zhejiang province  (East China) | 0.575 | 0.750 |
| BAM158 | Qingyao gourd | unclear | 0.792 | 0.679 |
| BAM161 | J002 | Anhui province  (East China) | 0.714 | 0.771 |
| BAM163 | G16 | Hunan province  (Central China) | 0.594 | 0.825 |
| BAM164 | J064 | Gansu province  (Northwest China) | 0.525 | 0.517 |
| BAM165 | G120 | Beijing province  (North China) | 0.625 | 0.557835 |
| BAM166 | J013 | Fujian province  (East China) | 0.600 | 0.825 |
| BAM167 | Yin-10 | Zhejiang province  (East China) | 0.104 | 0.113 |

**Supplementary Table 2|** Primer sequences used for qRT-PCR

| Primer Name | Sequence (5'-3') | Length (bp) |
| --- | --- | --- |
| *HG_GLEAN_10011803*-F | TTGAAGGTTGGGAGGTACATG | 122 |
| *HG_GLEAN_10011803*-R | ATGAAGTGGTACAGATGGCG |  |
| *HG_GLEAN_10001030*-F | GCTGCAACAGCTTACGATGA | 94 |
| *HG_GLEAN_10001030*-R | AGAGGGTGGAGGTGGATTTG |  |
| *HG_GLEAN_10001042*-F | CGGCTGCTCGTATCACAAAT | 117 |
| *HG_GLEAN_10001042*-R | TTGAGACAGCACTCGAGGAG |  |
| *HG_GLEAN_10001044*-F | TGTAGCTGGTGTTGAAGTACG | 146 |
| *HG_GLEAN_10001044*-R | ACTCCTGCATATTGGGTCTTC |  |
| *TuB-α*-F | AGCGTACCATTCAGTTTGTTGAT | 150 |
| *TuB-α*-R | AGACCTCAGCAACACTGGTAGAG |  |

**Supplementary Table 3|** Significant markers associated with FW resistance in at least one environment

| Env. | Marker | Chr. | Pos. | -log_10_ *P* | R^2^ (%) | Favored Allele |
| --- | --- | --- | --- | --- | --- | --- |
| DI2019 | BGReSe_14202 | 9 | 13,457,203 | 2.49 | 14.14 | G |
|  | BGReSe_13708 | 9 | 8,496,757 | 2.31 | 14.91 | C |
|  | BGReSe_12911 | 8 | 11,449,774 | 2.16 | 10.14 | G |
|  | BGReSe_12338 | 8 | 6,378,304 | 2.23 | 15.03 | T |
|  | BGReSe_09059 | 6 | 6,358,682 | 2.00 | 8.52 | A |
|  | BGReSe_07883 | 5 | 12,035,930 | 2.05 | 13.31 | G |
|  | BGReSe_04238 | 3 | 16,693,103 | 2.01 | 8.54 | T |
|  | BGReSe_03499 | 2 | 24,635,341 | 2.02 | 14.44 | G |
|  | BGReSe_02108 | 2 | 12,417,989 | 2.37 | 12.60 | G |
|  | BGReSe_01737 | 2 | 784,623 | 2.21 | 13.97 | C |
|  | BGReSe_01042 | 1 | 14,684,871 | 2.19 | 11.06 | C |
|  | BGReSe_00818 | 1 | 12,140,445 | 2.02 | 12.26 | T |
| DI2020 | BGReSe_14212 | 9 | 13,476,930 | 2.08 | 9.76 | G |
|  | BGReSe_14202 | 9 | 13,457,203 | 2.46 | 13.90 | G |
|  | BGReSe_12911 | 8 | 11,449,774 | 2.10 | 10.31 | G |
|  | BGReSe_05941 | 4 | 12,071,538 | 2.42 | 13.60 | T |
|  | BGReSe_05382 | 3 | 28,668,323 | 2.35 | 15.40 | G |
|  | BGReSe_05152 | 3 | 26,474,287 | 2.07 | 13.69 | C |
|  | BGReSe_02569 | 2 | 15,601,788 | 2.03 | 16.19 | A |
|  | BGReSe_02557 | 2 | 15,443,442 | 2.30 | 12.05 | G |
|  | BGReSe_02556 | 2 | 15,441,032 | 2.23 | 10.54 | G |
|  | BGReSe_02108 | 2 | 12,417,989 | 2.02 | 11.03 | G |
|  | BGReSe_01042 | 1 | 14,684,871 | 2.49 | 12.83 | C |
| aDI | BGReSe_14212 | 9 | 13,476,930 | 2.10 | 8.82 | G |
|  | BGReSe_14204 | 9 | 13,457,391 | 2.21 | 10.49 | G |
|  | BGReSe_14202 | 9 | 13,457,203 | 2.81 | 14.66 | G |
|  | BGReSe_12911 | 8 | 11,449,774 | 2.36 | 10.33 | G |
|  | BGReSe_12338 | 8 | 6,378,304 | 2.12 | 12.87 | T |
|  | BGReSe_05941 | 4 | 12,071,538 | 2.03 | 9.88 | T |
|  | BGReSe_05382 | 3 | 28,668,323 | 2.14 | 12.25 | G |
|  | BGReSe_02569 | 2 | 15,601,788 | 2.14 | 15.38 | A |
|  | BGReSe_02108 | 2 | 12,417,989 | 2.32 | 11.28 | G |
|  | BGReSe_01042 | 1 | 14,684,871 | 2.55 | 12.30 | C |
|  | BGReSe_00818 | 1 | 12,140,445 | 2.25 | 12.84 | T |
